# Effect of Enzymatic Hydrolysis on the Zinc Binding Capacity and *in vitro* Gastrointestinal Stability of Peptides Derived From Pumpkin (*Cucurbita pepo L*.) Seeds

**DOI:** 10.3389/fnut.2021.647782

**Published:** 2021-03-31

**Authors:** Dan Lu, Mengyao Peng, Min Yu, Bo Jiang, Hong Wu, Jingjing Chen

**Affiliations:** ^1^State Key Laboratory of Food Science and Technology, Jiangnan University, Wuxi, China; ^2^Key Laboratory of Agro-Products Processing, Institute of Agro-Products Processing Science and Technology, Xinjiang Academy of Agricultural and Reclamation Science, Shihezi, China

**Keywords:** pumpkin seed protein, peptides, chelating, zinc, gastrointestinal stability

## Abstract

Zinc is a crucial micronutrient for maintaining body immune system and metabolism function. However, insufficient intake from diet may lead to zinc deficiency and impair normal body function. In addition, conventional zinc salts supplementation has the disadvantage of low bioavailability since the zinc ions may be easily chelated by dietary fiber or phytate commonly found in diets rich in plants, and form precipitates that cannot be absorbed. Therefore, the objective of the present study is to prepare pumpkin seed derived peptides and to evaluate the effect of structure and surface properties on the zinc binding behavior of the pumpkin seed protein hydrolysate (PSPH), as well as their gastrointestinal stability. Briefly, different PSPHs were prepared using enzymatic hydrolysis method with bromelain, papain, flavourzyme, alcalase, and pepsin. The particle size, zeta potential, surface hydrophobicity, degree of hydrolysis, ATR-FTIR spectra, and zinc binding capacity were determined. The representative samples were chosen to characterize the binding energy and surface morphology of PSPH-Zn. At last, the *in vitro* gastrointestinal stability of PSPH and PSPH-Zn were evaluated. Our results showed that peptides hydrolyzed by papain had the largest average molecular weight, smallest particle size, highest hydrophobicity, and the greatest zinc binding capacity. Zinc showed better gastrointestinal stability in PSPHs chelates than in its salt. Meanwhile, PSPH-Zn with higher zinc binding capacity showed better stability. The result of this study indicated pumpkin seed hydrolyzed by papain may be used as a potential source for zinc fortification. The findings in this study may provide important implications for developing plant-based zinc chelating peptides.

## Introduction

Zinc, as one of the essential micronutrients with important structural, immune, and regulatory functions, contributes to the synthesis of the over 300 enzymes in the human body (1). A recent study found individuals with zinc deficiency may have higher COVID-19 infection risk (2). In addition, zinc plays a crucial role in the growth of infants, children, and adolescents. Zinc deficiency has a negative effect on growth retardation, anemia, and neuronal dysfunctions (3). Zinc deficiency is still quite common nowadays. It has been reported that about 4 to 73% of people in different countries still suffer from zinc deficiency, which may affect as many as two billion people worldwide (3).

Low dietary zinc intake and high loss of zinc during absorption can result in zinc deficiency, and may increase the risk of alopecia and skin rash (4). For this reason, different types of zinc supplements have been developed. In earlier years, zinc salts including zinc sulfate and zinc gluconate have been used as a zinc fortifier. However, phytate, a strong zinc chelator commonly existing in human diets, may form the insoluble complexes with zinc that cannot be absorbed (5). In the meantime, due to the lack of phytate-hydrolyzing enzyme, zinc could not be absorbed in the gastrointestinal tract. Furthermore, other minerals such as iron, calcium, and copper in food would compete for the non-specific divalent mineral transporters, affecting zinc absorption (6). Zinc salts supplements often have unpleasant metallic off-flavor, therefore compromising consumer acceptability.

The bioaccessibility and bioavailability of zinc are immensely affected by dietary zinc intake and absorption. Compared with inorganic zinc salts, amino acids or peptide binding can protect zinc from forming complexes with other dietary inhibitors, which will increase the digestion stability of zinc in the gastrointestinal tract and finally improving its bioavailability (4). Studies have showed that bioavailability of zinc is higher for zinc-peptide complexes than its inorganic counterparts (7). In recent years, zinc chelating peptide has been prepared from different protein sources, such as dairy (8), wheat germ protein (9), hemp (10), walnut (11), sesame (12), rapeseed (13), as well as sea cucumber (14), silver carp (15), octopus scraps (16), and tilapia bone (17).

Pumpkin (*Cucurbita pepo L*.) seed is a good source of protein (30–40%) (18). According to FAO/WHO, pumpkin seed protein is rich in all essential amino acids. It is a good source of valine, histidine, isoleucine, leucine, threonine, and methionine (19). In addition, pumpkin seeds have been recommended as a good source for obtaining dietary zinc by the World Health Organization (20). The literature findings have consistently shown that the total amount of Zn found in pumpkin seeds is about 91.2 μg·g^−1^ (20, 21). We therefore would like to explore whether the unique composition of pumpkin seed protein also makes it a good source for zinc-chelating peptides.

In this study, pumpkin seed proteins were hydrolysated by five different emzymes to prepare zinc-chelating peptides. Further, the surface hydrophobicity, zeta potential, particle size, molecular weight distribution, and amino acid composition of hydrolysis were characterized. Meanwhile, the complex of zinc and peptide were characterized by SEM, XPS, and FT-IR. The *in vitro* gastrointestianl stability of zinc was then evaluated using simulated gastrointestinal digestion. The findings of this study would be of prime importance for the utilization of pumpkin seed protein to fabrication zinc-chelating peptides, which may be used as a functional food ingredient or used in plant-based food to realize the fortification of zinc.

## Materials and Methods

### Materials

Pumpkin seeds (*Cucurbita pepo L*.) were provided by Haichuansanxin Food Company (Beitun, China). Alcalase (≥200 U/mg) was purchased by Hefei Bomei Biotechnology Co., Ltd. (Hefei, China). Papain (>200 units/mg) and flavourzyme (30,000 units/g) were purchased from Solarbio Life Science (Beijing, China). Bromelain (3,000–7,000 U/mg), pepsin (1,200 U/g), pancreatin (≥4,000U/g), trypsin (≥50,000U/g), and bile salt and o-Phthalaldehyde (OPA) and dithiothreitol (DTT) were purchased from Sangon Biotech (Shanghai, China). In addition, 8-Anilino-1-naphthalenesulfonic acid (ANS) [≥97% (HPLC)] was purchased from Sigma-Aldrich. Sodium dodecyl sulfate (SDS) and L-Serine (≥BR) were purchased from Sinopharm Chemical Reagent Co., Ltd. Zinc sulfate heptahydrate, sodium hydroxide, nitric acid, n-hexane, ethyl alcohol, and other reagents were of analytical grade and purchased from Chemical Reagent Co., Ltd. (Shanghai, China). Dialysis bags (MD34-500) were purchased from Wuxi Hengkang Medical Technology Co., Ltd.

### Preparation of Samples

#### Preparation of Pumpkin Seed Protein

The pumpkin seeds were ground into flour using a YF-1000 blender (Yongli Pharmaceutical Mechanic, Zhejiang, China). Ten volumes of hexane (w/v) were added to pumpkin seed flour to remove fat. After stirring for 2 h, the hexane was removed. This process was repeated three times to ensure complete removal of fat. The defatted pumpkin seed flour was dispersed in deionized water (w/v:1/10) and adjusted pH 10 with 1 M NaOH. Then the mixture was stirred gently for 30 min and centrifuged at 4°C and 10,000 g for 20 min. The supernatant was collected and then filtered with a filter paper. The protein dissolved in the filtrate was sedimented by adjusting pH to 5.00 with 1 M HCl. The precipitation was washed with deionized water, centrifuged at 10,000 g for 20 min to remove possible impurities. The pH of the protein was adjusted to 7 using NaOH. Then it was frozen-dried to obtain pumpkin seed protein powder. The powder was stored in −20°C for further use.

#### Preparation of Pumpkin Seed Protein Hydrolysates

The pumpkin seed protein powder was dissolved in deionized water (4%, w/w). Pumpkin seed protein solution was boiled in a water bath for 15 min to inactivate endogenous enzymes. After cooling, the pH was adjusted with 1 M NaOH or 1 M HCl for enzymatic hydrolysis. Thereafter, the solutions were hydrolyzed with bromelain (pH 6.5, 37°C), alcalase (pH 8.0, 50°C), papain, flavourzyme (pH 7.0, 55°C), papain (pH 7.0, 55°C), and trypsin (pH 7.5, 37°C) (2% enzyme to substrate ratio, w/w) for 3 h. Each protein solution was placed in a conical flask with sealing membrane and was hydrolyzed in constant-temperature shaker water bath using the water-bathing Constant Temperature Vibrator (THZ-82, Changzhou Guohua Electric Appliance Co. Ltd). After the enzymatic hydrolysis, the enzyme was deactivated by heating in a boiling water bath for 15 min and cooled to room temperature. The pH was adjusted to 7 by 1 M NaOH or 1 M HCl and centrifuged at 10,000 g for 15 min. The precipitation was discarded, and the supernatant was retained. The supernatant was filtered and freeze dried. Then the pumpkin seed protein hydrolysate (PSPH) was obtained and stored in a −20°C freezer for later use.

#### Preparation of PSPH-Zn

Peptide-zinc complexes were prepared in reference to previous methods with some modifications (10). PSPHs were respectively dissolved in the deionized water at the concentration of 20 mg/ml. Then, 100 mM zinc sulfate solution was dripped into the peptide solution slowly to make the final concentration of peptide: zinc to 1:1 (g: mmol), and the mixtures were incubated in 60°C water shaking bath for 60 min. After that, ethanol was added into the incubates until the ethanol concentration was up to 80% to precipitate peptides and peptide-zinc (II) complexes. Left to stand for 60 min at room temperature, the intermixture was centrifuged at 10,000 g for 10 min to get the centrifugal precipitates. The white precipitates were washed three times with 80% ethanol to remove the unbound zinc. Finally, the peptide–zinc (II) complexes were collected and prepared for freeze-dried structure characterization.

### Characterization of PSPH and PSPH-Zn

#### Degree of Hydrolysis

The degree of hydrolysis (DH) of PSPHs were determined using the o-Phthalaldehyde (OPA) method described by Nielsen et al. (22). The PSPH was dissolved in deionized water, and the 400 μl sample solutions was mixed with 3 ml OPA-reagents for 2 min precisely. Then the optical density (OD) was measured at 340 nm using an UV-Visible Spectrophotometer (MAPADA P7, Shanghai, China). Double distilled water was used as blank. The degree of hydrolysis was calculated using the following equations:

$$\begin{array}{l} Serine\;NH_{2}=\frac{OD_{sample}-OD_{blank}}{OD_{standard}-OD_{blank}}*\frac{0.9516meqv}{L}*\frac{d}{c} \end{array}$$

where serine-NH_2_ represents meqv serine NH_2_/g protein, OD_sample_ was the absorbance of each sample, the OD_standard_ was the absorbance of serine standard, *d* was dilution factor, and c was protein content of pumpkin seed.

$$\begin{array}{l} DH\left(\%\right)=\frac{h}{h_{tot}}*100\%=\frac{\frac{Serine\;NH_{2}*\beta }{\alpha }}{h_{tot}}*100\% \end{array}$$

where h was number of hydrolyzed peptide bonds, α = 1, β = 0.4, h_tot_ is the total number of peptide bonds in the protein substrate (meqv/g protein).

#### Molecular Weight Distribution

The molecular weight distribution of PSPHs was determined by gel permeation chromatography (GPC) using a high performance liquid chromatography (HPLC) system (Agilent 1100, Agilent Technologies Inc., Germany). Chromatographic column: TSK gel SWXL 300 mm × 7.8 nm; 2 ml sample solution was added into a 10 mL volumetric flask, acetonitrile/water/trifluoroacetic acid (45/55/0.1: v/v/v) solution was added to bring the volume to 10 ml. The solution was then filtered and analyzed on the HPLC. The flow rate was 0.5 ml/min. The temperature of the column was 30°C and the sample was detected at 220 nm.

#### Zeta Potential and Average Particle Diameter Analysis

Dried PSPH (0.5%, w/v) was dissolved in deionized water and diluted 10 times for measurement. The mean particle diameter and zeta potential of PSPHs were determined using a Zetasizer (Nano-ZS, Malvern Instruments Ltd., Malvern, UK).

#### Surface Hydrophobicity

The surface hydrophobicity of PSPHs was analyzed using 8-Anilino-1-naphthalenesulfonic acid (ANS) as fluorescent probe. PSPH was dissolved (0.05%, w/v) in phosphate buffer (10 mM, pH 7.0). Each protein dispersion was diluted with phosphate buffer to obtain serial protein concentrations of 0.005 to 0.025% (w/v). Then, 20 μl ANS solution (8.0 mmol/L) was added into 4 ml protein solution and mixed thoroughly. The samples were measured using a Hitachi F-7000 fluorescence spectrophotometer (Hitachi Lt., Tokyo, Japan). The excitation wavelength was 390 nm and an emission wavelength was 470 nm. The fluorescence intensity was plotted on the ordinate and the protein concentration was used to the abscissa. The initial slope was used as an index of protein hydrophobicity (*H*_0_).

#### Amino Acid Composition Analysis

The amino acid composition was determined in reference to reported methods (23). Dried PSPH was digested using 6 M HCl at 110°C for 24 h under nitrogen atmosphere. The composition of amino acid was measured by Sykam S433D automatic amino acid analyzer (Munich, Germany).

#### Zinc Binding Capacity

Zinc binding capacity was calculated according to the method published by Wang (10). The samples were digested in nitric acid using a hot plate at 150°C for 2 h and 180°C for 1 h until smoke was observed. After cooling, the digestion solution was transferred into a 10 ml or 25 ml volumetric flask and brought to volume with H_2_O. After further dilution of 1,000 to 2,000-fold, the content of Zn^2+^ was measured using atomic absorption method (Atomic Absorption Spectrometer, Thermo Fisher Scientific). Zinc binding capacity was calculated as described previously using the following formula:

$$\begin{array}{l} Zinc\;binding\;capacity\;\left(\%\right)\\ =\frac{The\;amount\;of\;Zn^{2+}\;in\;the\;complex\;\left(mg\right)\;}{The\;amount\;of\;Zn^{2+}\;added\;\left(mg\right)}*100\% \end{array}$$

#### Attenuated Total Reflection Fourier Transform Infrared Spectroscopy (ATR-FTIR)

The ATR-FTIR spectra of PSPHs (PSPH-Bro, PSPH-Alc, PSPH-Fla, PSPH-Pap, PSPH-Try) were determined using a Nicolet iS10 FTIR spectrophotometer (Thermo Fisher Scientific Corp., Waltham, USA). Each sample was placed on the crystal and the data was collected. The ATR-FTIR spectra was scanned 16 times with a spectral resolution of 2 cm^−1^ and recorded at the wavenumbers between 4,000 to 400 cm^−1^. The data was processed with the OMNIC software (Thermo Fisher Scientific Inc., OMNIC 9.2.86).

#### Morphology of PSPH and PSPH-Zn Complexes

A scanning electron microscopy (SEM, S-4800, Hitachi Science Systems, Ltd., Tokyo, Japan) was used to observe detailed surface morphology of representative PSPH and PSPH-Zn complex. The powders were glutted on the plate, respectively, sprayed with gold, and then observed using the SEM at an accelerating potential of 5 kV.

#### X-Ray Photoelectron Spectroscopy Spectra of PSPH and PSPH-Zn Complexes

X-ray photoelectron spectroscopy measurements of representative PSPH and PSPH-Zn were carried out with an AXIS Supra by Kratos Analytical Inc. (Wharfside, Manchester, UK). The monochromatized Al Ka radiation (hv = 1,486.6 eV, 225 W) was used as an X-ray source. The base pressure is 10^−9^ torr. A pass energy of 160 eV and a 1 eV step size was used to obtain survey scan spectra. A pass energy of 40 eV and a 0.1 eV step size was used to obtain narrow region scans. The analyzed area of all XPS spectra was 300 × 700 μm^2^. A charge neutralizer was used throughout as the samples were mounted so that they were electrically isolated from the sample bar. All spectrums were calibrated using C 1s (284.8 eV).

#### Gastrointestinal Stability of PSPH and PSPH-Zn

The gastrointestinal stabilities of PSPH and PSPH-Zn were determined using the methods reported by Udechukwu et al. (8) and Liao et al. (17) with some modifications. Briefly, the simulated gastric juice was prepared by pepsin in 0.1 M hydrochloric acid. And the pancreatin and bile salts were dissolved in 0.1 M NaHCO_3_ solution to prepare simulated intestinal juice. First, the PSPH-Zn complexes were dissolved in Milli-Q water and incubated with simulated gastric juice at pH 2.0 and 37°C for 30 min in the linear shaking bath. The pepsin-substrate ratio was 1:100 (w/w). After incubation, a part of gastric digests was withdrawn and heated for 10 min in boiling water to inactivate enzymes. Then, the solutions were dialyzed in dialysis bags (molecular weight: 34–500 Da) for 6 h. Thereafter, the contents of zinc in dialysis bags were measured by atomic absorption spectrometry. Another portion of the digests were adjusted the pH to 7.5 with 1 M NaOH and continuously reacted with intestinal juice maintained the pH at 37°C for 3 h. The intestinal digests were treated with the same operation as the gastric digests. Meantime, the PSPH were used as the control. The zinc stability (%) was expressed as the percentage of the total zinc retained after dialysis.

### Statistical Analysis

The experimental results were expressed in the form of mean ± standard deviation (Means± SD). The statistical analysis was conducted using the software (IBM SPSS statistic 22). One-way variance analysis ANOVA followed by Duncan's multiple comparison test was used to detect the difference between the mean values, and the *P* < 0.05 was considered as significantly different.

## Results

### Molecular Weight Distribution and Degree of Hydrolysis of PSPHs

**Figure 1:**
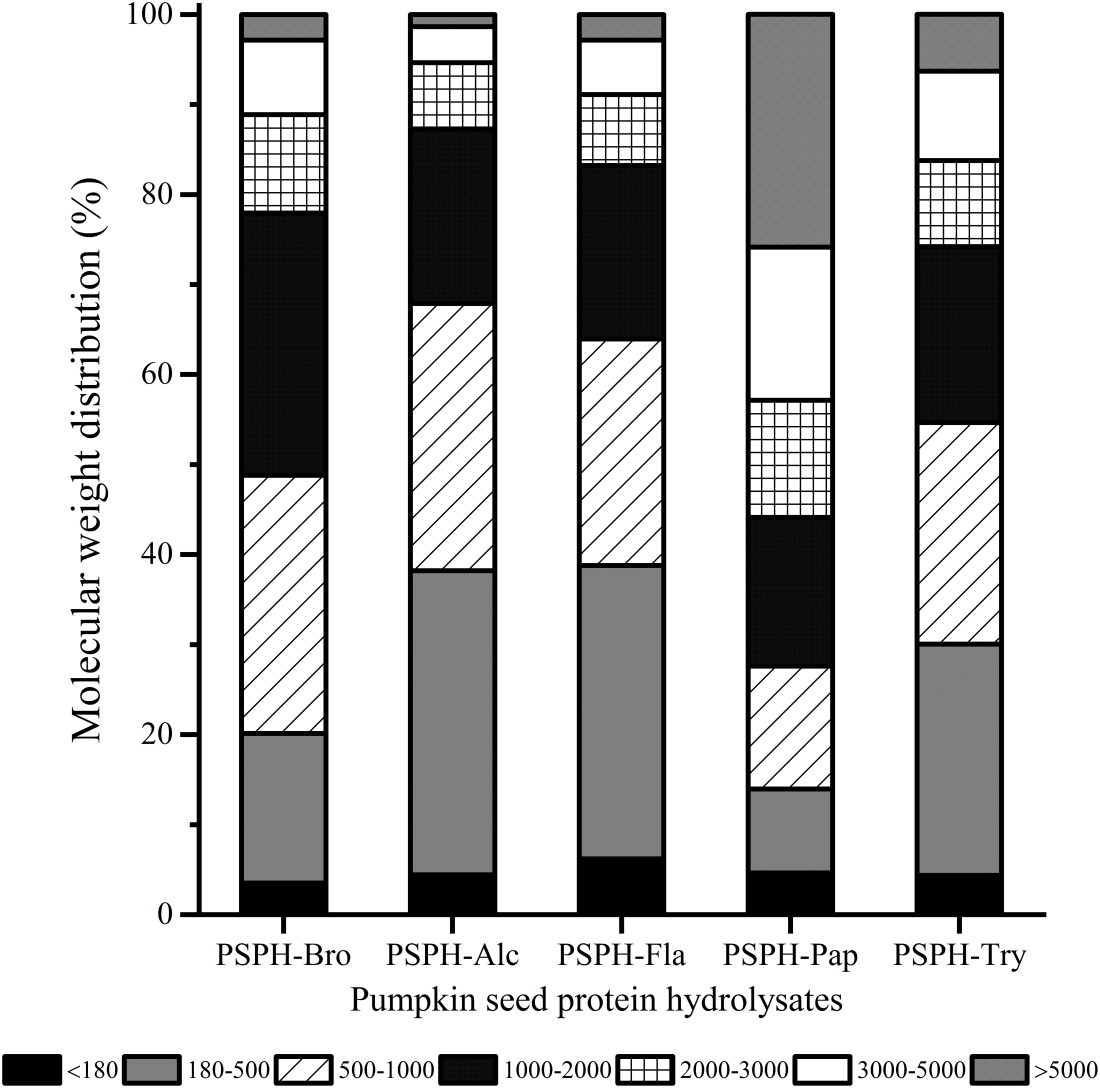
Molecular weight distribution of pumpkin seed protein hydrolysates produced with bromelain (PSPH-Bro), alcalase (PSPH-Alc), flavourzyme (PSPH-Fla), papain (PSPH-Pap), trypsin (PSPH-Try) after hydrolysis 3 h in a 2% (w/w) enzyme to substrate ratio.

The relationship between the molecular weight distribution of hydrolysates and their mental-binding properties was still not fully understood, which may differ when different peptides were derived from different sources. Chen at al. found that compared with other two peptides with different average molecular weights (P2: 2,745 Da, P3: 4,378 Da), peptide with low molecular weight (P1:1,653 Da) had stronger zinc chelation capacity and stability (24). In addition, chicken muscle peptides (>10 kDa) showed excellent chelation capacity with iron (25). The molecular weight distribution of different enzymatic hydrolysates at the same hydrolysis time (3 h) could be clearly seen from Figure 1. The profile of different molecular hydrolysates was divided into 6 groups (<180 Da, 180–500 Da, 500–1,000 Da, 1,000–2,000 Da, 2,000–3,000 Da, and >3,000 Da). The mean molecular weights of PSPH-Bro, PSPH-Alc, PSPH-Fla, PSPH-Pap, and PSPH-Try were 1,463 Da, 1,022 Da, 1,167 Da, 3,312 Da, and 1,582 Da, respectively. The molecular weight (Mw) of PSPH-Pap >3,000 Da accounted for more than 50%, while the percentage of molecular weight smaller than 1,000 Da was the lowest compared with other hydrolysates. And, from **Figure 5**, the PSPH-Pap has the highest zinc binding capacity compared with other hydrolysates. These results indicated that, for pumpkin seed peptides, high molecular hydrolysates can better bind with zinc ions.

**Figure 2:**
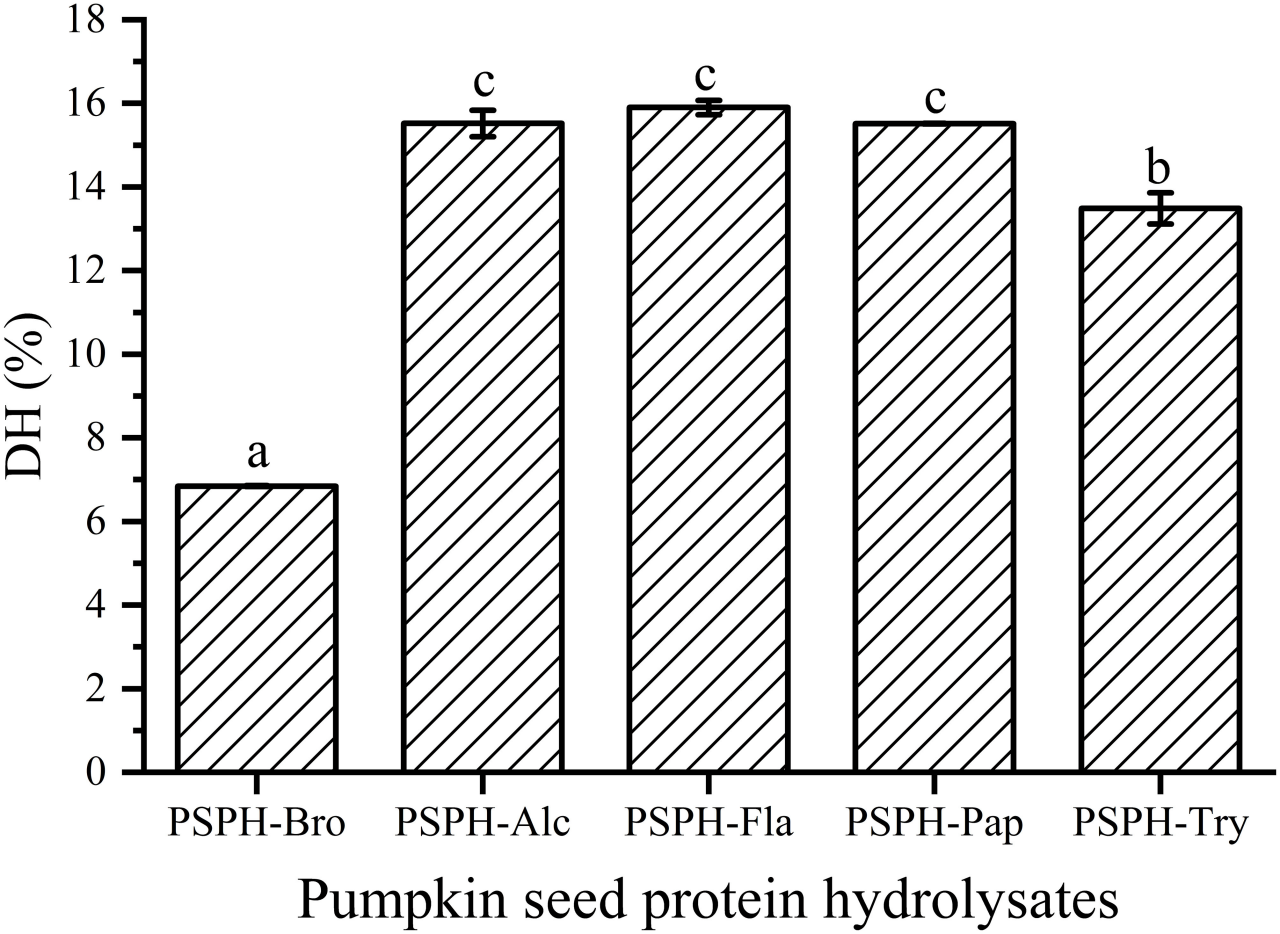
Degree of hydrolysis (DH) of pumpkin seed protein hydrolysates produced with bromelain (PSPH-Bro), alcalase (PSPH-Alc), flavourzyme (PSPH-Fla), papain (PSPH-Pap), trypsin (PSPH-Try) after hydrolysis 3 h in a 2% (w/w) enzyme to substrate ratio. Different letters represent significantly different mean values with *P* < 0.05.

DH was another factor affecting the amino acid components and sequences, which may further influence the zinc-binding capacity. There had been reports that the degree of hydrolysis was positively correlated with mental-binding capacity. Sun et al. (26) reported that sea cucumber (*Stichopus japonicus*) ovum hydrolysates produced with alcalase showed the best DH and the highest iron-binding capacity. In our study, all hydrolysates were hydrolyzed with their respective optimum pH and temperature for 3 h. Degree of hydrolysis of the five hydrolysates were 6.8 ± 0.1% for PSPH-Bro, 15.5 ± 0.3% for PSPH-Alc, 5.9 ± 0.2% for PSPH-Fla1, 15.5 ± 0.2% for PSPH-Pap, and 13.5 ± 0.4% for PSPH-Try (Figure 2). The DH of four hydrolysates were almost identical around 15%. The PSPH-Pap showed the greatest zinc binding capacity though the degree of hydrolysis was lower than that of alcalase and flavourzyme. The similar results can be found in the study of Wu et al. (27). DH was an influential factor affecting mental-binding capacity; a too high or too low DH is inappropriate for mental binding (28). The essential reason of the phenomenon was that proper hydrolysis was beneficial to the exposure of hydrophobic groups, which contributed to the coordination of mental and peptides (29).

### Average Particle Sizes and Zeta Potential of PSPHs

**Figure 3:**
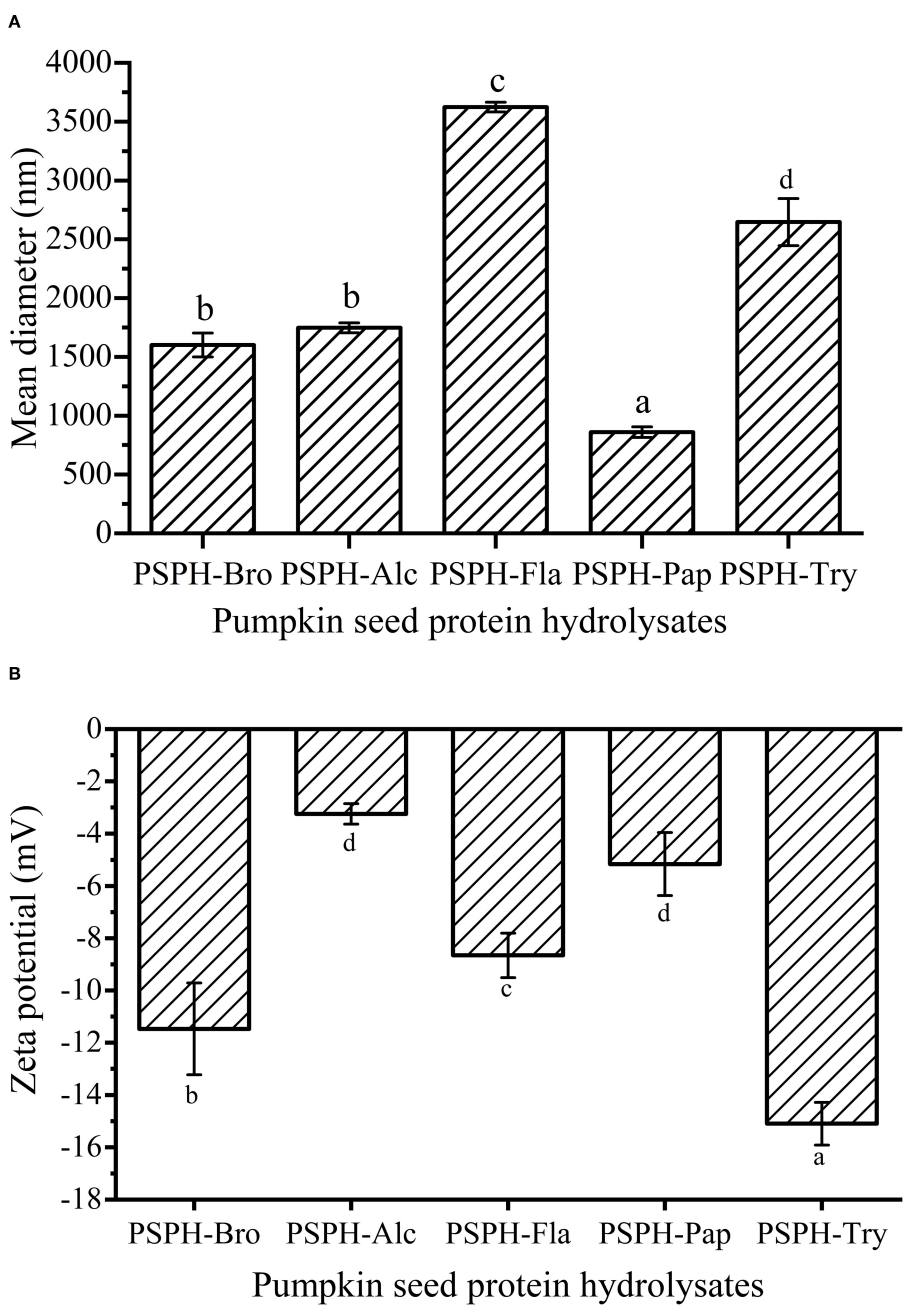
Mean particle diameter **(A)** and Zeta potential **(B)** of pumpkin seed protein produced with bromelain (PSPH-Bro), alcalase (PSPH-Alc), flavourzyme (PSPH-Fla), papain (PSPH-Pap), trypsin (PSPH-Try) after hydrolysis 3 h in a 2% (w/w) enzyme to substrate ratio. Bars in each chart with different letters represent significantly different mean values with *P* < 0.05. Different letters represent significantly different mean values with *P* < 0.05.

Particle size of a carrier system played an important role in the bioaccessibility and bioavailability of the targeted compound it encapsulates (30). In most cases, the peptides possessing smaller sizes demonstrate better solubility and contributed to better digestibility of zinc compared with intact proteins (7). As shown in Figure 3A, the average particle diameters of PSPH-Bro, PSPH-Alc, PSPH-Fla, PSPH-Pap, and PSPH-Try were 1,601 ± 101 nm, 1,747 ± 42 nm, 3,624 ± 41 nm, 859 ± 45 nm, and 2,646 ± 200 nm, respectively. The PSPH-Fla and PSPH-Try had relatively larger average particle sizes, while the mean particle size of PSPH-Pap was the smallest. In most cases, the peptides possessing smaller sizes demonstrate better solubility and contributed to better digestibility of zinc compared with intact proteins (7).

The Zeta potential of the particles was defined as the electric potential at the boundary of the double layer on the particle surface (31). According to the Udechukwu report, the zinc-chelating capacity of the whey protein hydrolysates had a significantly strong negative relationship with their ζ-potential (8). The five pumpkin seed protein hydrolysates were all negatively charged, which could be observed at Figure 3B, and the zeta potential of PSPH-Bro, PSPH-Alc, PSPH-Fla, PSPH-Pap, and PSPH-Try were −11.47 ± 1.76 mV, −3.24 ± 0.39 mV, −8.66 ± 0.85 mV, −5.16 ± 1.21 mV, and −15.1 ± 0.82 mV. The PSPH-Alc and PSPH-Pap showed relatively low potential. The difference in zeta potential difference was caused by the specific cleavage behavior of different enzymes. For example, papain can cleave the bond between arginine/lysine and non-valine amino acid, releasing peptides with arginine/lysine as terminal amino acid, and leading to the change of zeta potential. As shown in Figures 3A,B, PSPH-Psp had the smallest mean particle diameter but its net charge was not the highest, different from the theory used in colloid systems where high net negative charge will result in smaller mean particle diameter caused by electric repulsion. It was probably because each PSPH is a complicated system containing different amounts of peptides with varied compositions, chain length, and conformation.

### Surface Hydrophobicity of PSPHs

**Figure 4:**
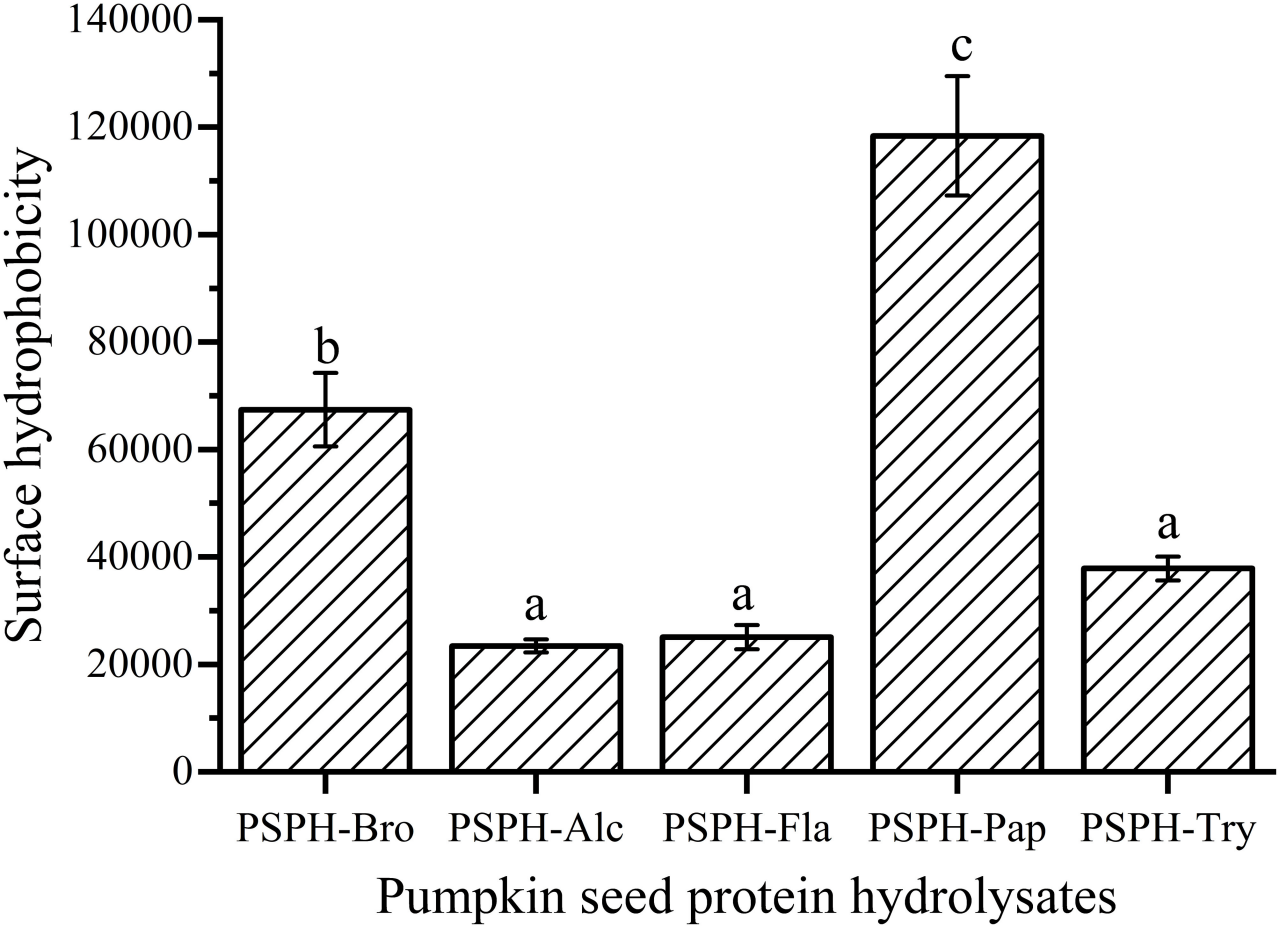
Surface hydrophobicity of pumpkin seed protein hydrolysates produced with bromelain (PSPH-Bro), alcalase (PSPH-Alc), flavourzyme (PSPH-Fla), papain (PSPH-Pap), trypsin (PSPH-Try) after hydrolysis 3 h in a 2% (w/w) enzyme to substrate ratio. Different letters represent significantly different mean values with *P* < 0.05.

**Figure 5:**
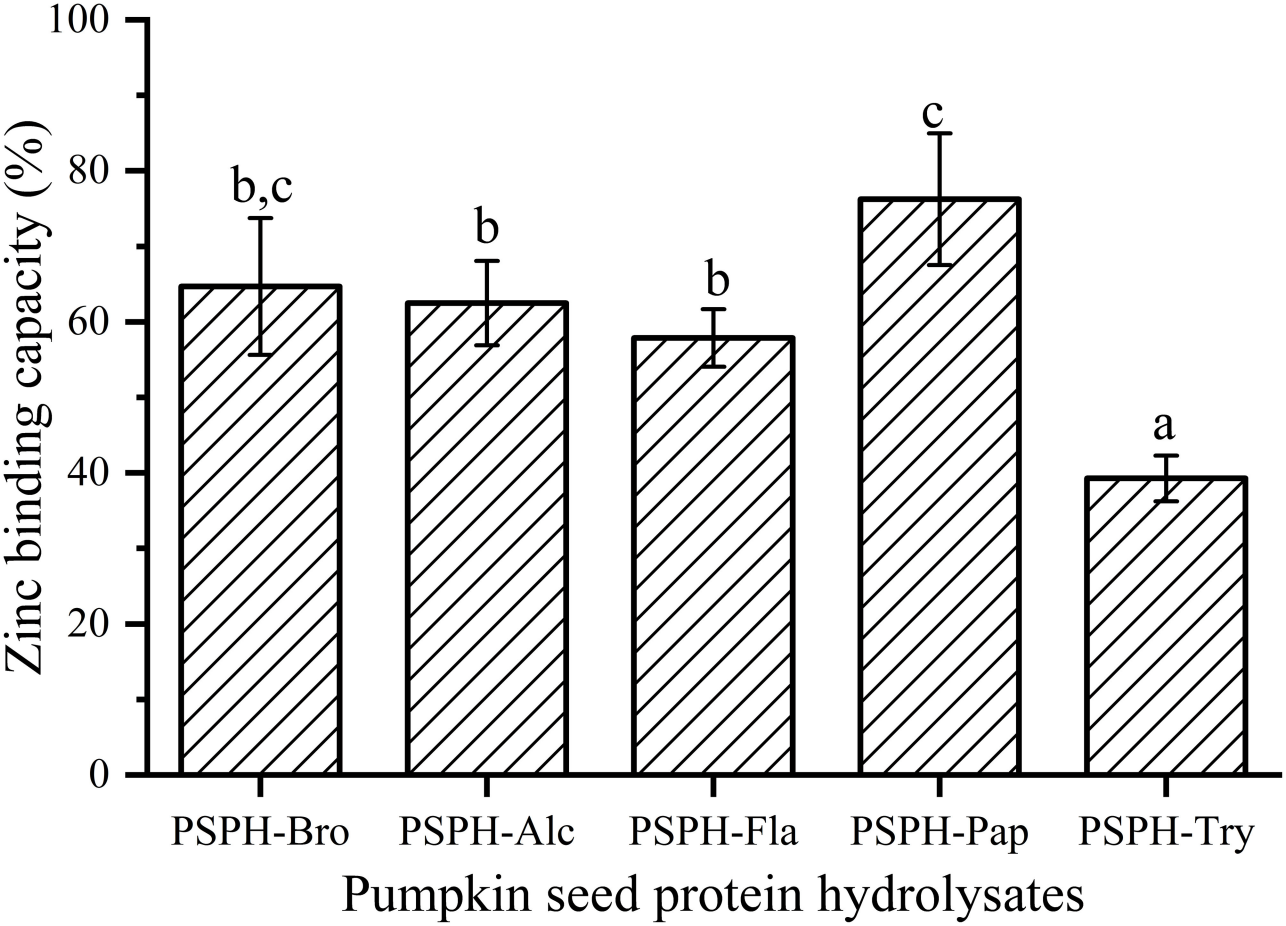
Zinc binding capacity of pumpkin seed protein hydrolysates produced with bromelain (PSPH-Bro), alcalase (PSPH-Alc), flavourzyme (PSPH-Fla), papain (PSPH-Pap), trypsin (PSPH-Try) after hydrolysis 3 h in a 2% (w/w) enzyme to substrate ratio. Different letters represent significantly different mean values with *P* < 0.05.

The surface hydrophobicity of PSPHs was shown in Figure 4. The PSPH-Pap had the highest surface hydrophobicity and PSPH-Alc and PSPH-Fla had lowest surface hydrophobicity. Combined with the results in Figure 5, the zinc binding capacity is basically positively correlated with surface hydrophobicity. The surface hydrophobicity of proteins was caused by the exposure of some hydrophobic groups on the surface of proteins. It was the main factor affecting the intermolecular interaction and one of the key indicators to measure the functional properties of proteins.

### Amino Acid Composition of PSPHs

The function of the peptide depends mainly on its composition and sequence of amino acid composition. In general, the side chain of histidine, aspartate, glutamate, cysteine, and serine can bind with minerals including Cu, Fe, and Zn through N, O, or S atoms (7). The imidazole group of histidine, the phenol ring of tyrosine, and the carboxy group of aspartate and glutamate can serve as the metal binding sites in proteins (32). Specifically, studies showed that zinc may bind to the acidic side chain of protein, phosphorylated residue, deprotonated nitrogen, and sulfur residues of amino acids and peptides. While functional groups including thiol group (cysteine, glutathione), imizadole group (hisidine), as well as asparagine and glutamine residues, phosphorylated residues, and methionine all contributed to the increased chelation capacities of zinc (33). The amino acid profiles of pumpkin seed protein and five enzymatic hydrolysates were shown in Table 1. PSPH-Pap contained more (45.45%) amino acids (Asp, Glu, His, Ser, Cys) that may chelate with zinc than other groups: 40.4% for native protein, 42.25% for PSPH-Bro, 42.58% for PSPH-Alc, 42.64% for PSPH-Fla, and 44% for PSPH-Try.

**Table 1:** Amino acid composition of pumpkin seed protein and pumpkin seed protein hydrolysates.

**Amino acid**	**PSP**	**PSPH-Alc**	**PSPH-Tyr**	**PSPH-Fla**	**PSPH-Pap**	**PSPH-Bro**
Asp	9.24	9.19	9.21	8.93	8.80	8.33
Glu	19.74	23.04	21.92	22.52	25.53	24.30
Ser	4.65	4.86	4.84	4.84	5.24	5.00
His	2.31	1.93	2.08	1.92	1.46	1.87
Gly	4.46	4.43	4.53	4.43	4.42	4.50
Thr	2.98	2.83	2.83	2.70	2.30	2.53
Arg	15.31	15.85	15.93	16.43	17.84	18.29
Ala	4.65	4.67	4.63	4.40	4.02	4.22
Tyr	3.74	3.37	3.31	2.94	2.50	3.13
Cys	0.49	0.52	0.56	0.52	0.48	0.56
Val	5.60	4.50	4.60	4.44	4.19	4.03
Met	2.46	2.33	2.36	2.23	1.85	2.20
Phe	5.60	5.19	5.39	5.28	4.63	4.68
Ile	4.51	4.29	4.38	4.33	3.61	3.81
Leu	7.58	7.04	7.38	7.23	6.28	6.50
Lys	3.61	3.46	3.66	3.61	3.63	3.46
Pro	3.07	2.51	2.37	3.23	3.23	2.60

*PSP, pumpkin seed protein*.

### Zinc Binding Capacity of Different PSPHs

Zinc binding capacity or zinc chelating capacity was defined differently according to different experiments. In this study, it was defined as the percentage of zinc that was bound to PSPHs to that of total zinc added to the mixture. As shown in Figure 5, the zinc binding capacity of PSPHs were 64.7 ± 9.1% for PSPH-Bro, 62.5 ± 5.6% for PSPH-Alc, 57.9 ± 3.8% for PSPH-Fla, 76.3 ± 8.7% for PSPH-Pap, and 39.3 ± 3.0% for PSPH-Try, respectively. Zinc binding capacity of a certain peptide is predominately determined by the amino acid composition and arrangement of that specific peptide (33). According to the above-mentioned results, PSPH-Pap had the largest average molecular weight of 3,312 Da and contained the highest content of amino acid that tend to chelate with zinc. Udechukwu also found there was a positive correlation between zinc chelating amino acid content and zinc chelating capacity (8). In this study, each PSPH is the mixture of protein hydrolysate with different chain length as well as amino acid composition. The zinc binding capacity of PSPHs are the collective manifestation of peptide structural and chemical properties including surface hydrophobicity, average molecular weight distribution, zeta potential, as well as amino acid composition.

### ATR-FTIR

**Figure 6:**
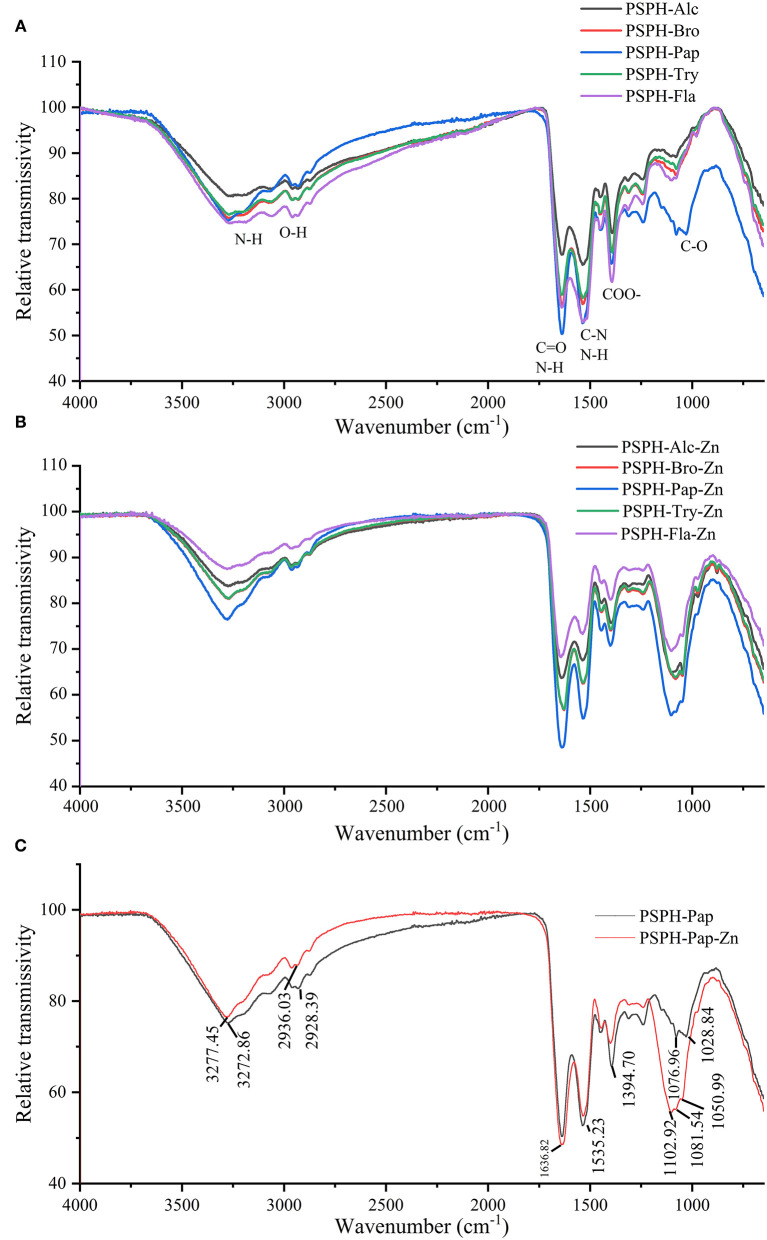
Attenuated total reflection fourier transform infrared spectra (ATR-FTIR) of **(A)** pumpkin seed protein hydrolysates (PSPHs) in the range of 4,000 to 400 cm^−1^; **(B)** pumpkin seed protein hydrolysates (PSPH) zinc chelates; **(C)** pumpkin seed protein hydrolysate hydrolyzed by papain (PSPH-Pap, black line) and its zinc complex (PSPH-Pap-Zn, red line).

In order to understand the ligand interactions between PSPH and Zn, ATR-FTIR was applied to characterize the structure of PSPH-Zn. The IR spectra of five hydrolysates of pumpkin seed protein and their zinc-binding complexes are shown in Figures 6A–C. The PSPH-Pap, as the peptide with the highest zinc chelation rate, was individually analyzed in detail with its zinc binding complex: PSPH-Pap-Zn.

The infrared spectra consist of two parts with no doubts: functional group region and fingerprint region. The band around 3,400 cm^−1^ represented the N-H stretching, while O-H bonds existed an absorption in the wavenumber of 2,900 cm^−1^. Furthermore, the band around 1,600 cm^−1^ exhibited the N-H bending vibration coupled with the C=O stretching, while the absorption at 1,400 cm^−1^ was due to COO- and C-O peak occurred in 1,100 cm^−1^. From Figure 6A, there were no obvious differences among the five hydrolysates.

As shown in Figure 6B, after binding to zinc, both transmissivity intensity and peak position were changed for peptide-zinc complex. Take PSPH-Pap with the highest zinc-chelating capacity, for example; the peaks slightly shifted from 3,272.86 cm^−1^ to 3,277.45 cm^−1^ and 2,928.39 cm^−1^ to 2,936.03 cm^−1^, which may be caused by the stretching vibrations of N–H bonds and O-H bonds. The most apparent peak feature could be observed around 1,100 cm^−1^, while the 1,076.96 cm^−1^, 1,028.84 cm^−1^ shifted to the 1,102.92 cm^−1^ and 1,081.54 cm^−1^. Moreover, the new absorption peaks appeared in 1,050.69 cm^−1^. The sharp peak observed around 1,400 cm^−1^ may be attributed to the stretch of C-O. In conclusion, oxygen atoms from C-O bonds and nitrogen-atoms from the N-H bonds were the main binding sites of PSPH and zinc.

### SEM

**Figure 7:**
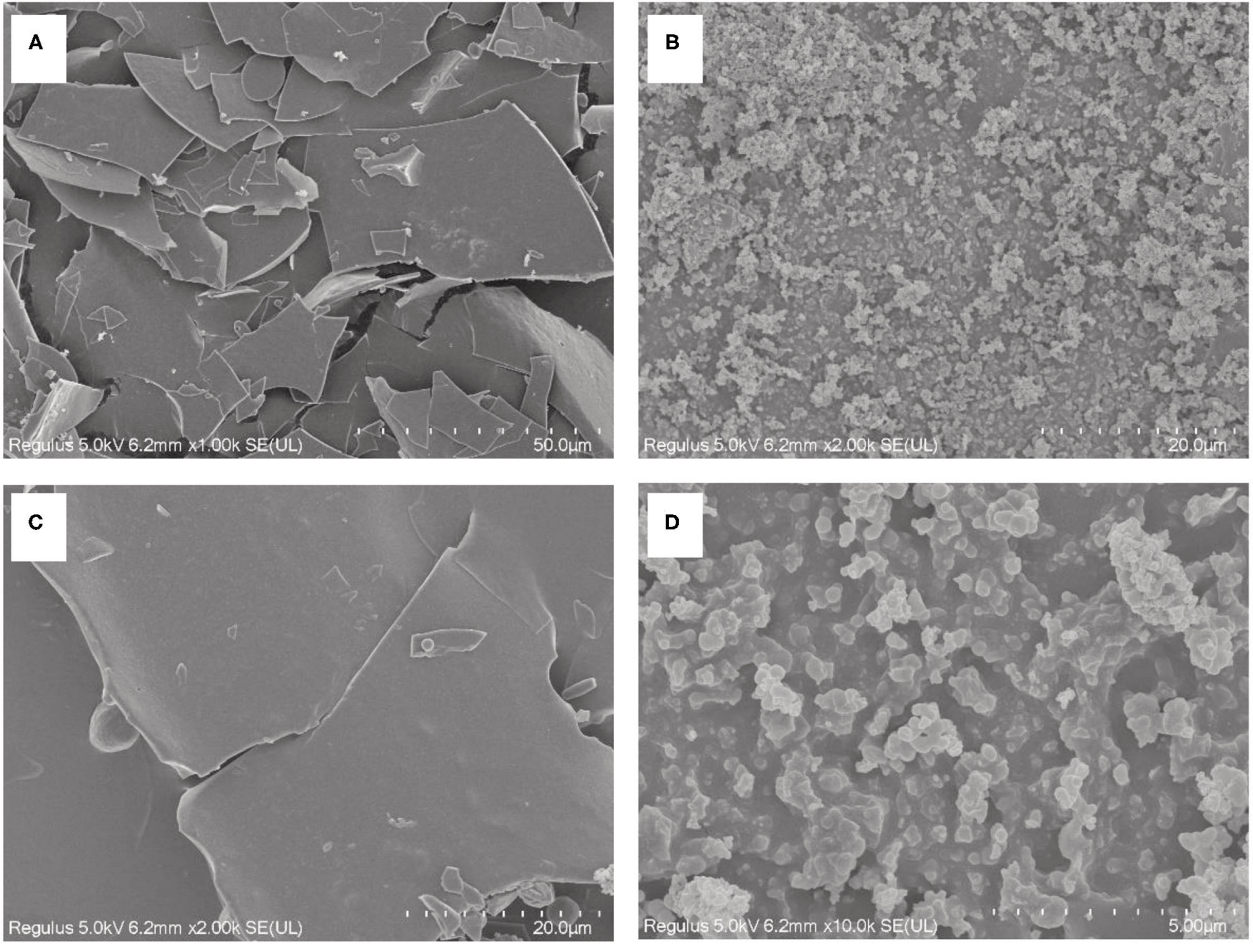
Scanning electron microscopy (SEM) photograph of pumpkin seed protein hydrolysates produced by papain after hydrolysis 3 h in a 2% (w/w) enzyme to substrate ratio PSPH-Pap **(A, C)** and its zinc complex PSPH-Pap-Zn complex **(B, D)**.

Binding to zinc has an influence on both the chemical structure and microstructure of PSPHs. Figure 7 showed the microstructures of pumpkin seed protein hydrolysate (PSPH), PSPH-Zn, respectively. The PSPH showed a laminar structure with smooth surface (Figures 7A,C). However, the morphology of PSPH-Pap-Zn seemed to be the loose globular structure (Figures 7B,D), which could be explained as the aggregation of peptide due to the existence of zinc. In the presence of zinc, the spatial conformation of the peptides may rearrange or fold where different ligands from the amino acid side chains forms complex with zinc through coordination (34). A similar phenomenon has also been observed in the oyster-derived peptide zinc-binding complex (35), cucumber seed peptide-calcium chelate (36). Zhang et al. (37) reported that the peptide could fold and aggregate when the oyster protein hydrolysis and zinc form the compound. A detailed understanding on the molecular conformation change after zinc chelation could be obtained through molecular modeling.

### X-Ray Photoelectron Spectroscopy Spectra of PSPH and PSPH-Zn

**Figure 8:**
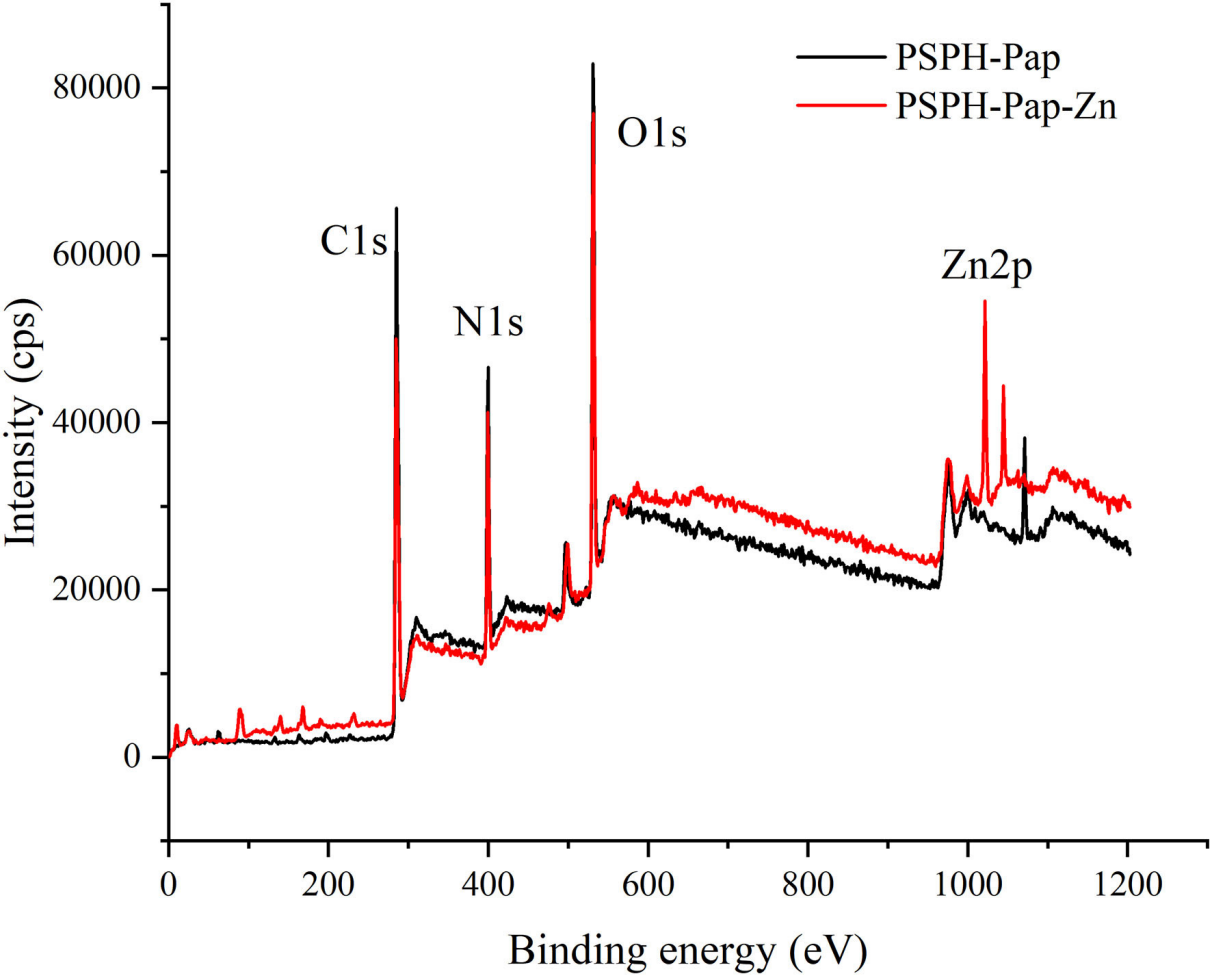
X-ray Photoelectron Spectroscopy (XPS) spectra of pumpkin seed protein hydrolysates produced by papain after hydrolysis 3 h in a 2% (w/w) enzyme to substrate ratio (PSPH-Pap, black line) and its zinc complex (PSPH-Pap-Zn, red line).

XPS is a method used to analyze the surface chemistry of a material. XPS instrument measures the kinetic energy emitted from surface elements upon X-ray exposure. It has been used to characterize the binding of iron to an Antarctic krill derived peptide (38). Since each of our PSPH samples is a mixture of peptides with different molecular weights, the XPS was only used quantitatively to identify the PSPH-Zn. As shown in Figure 8, there were three peaks of C1s, N1s, O1s at 284.8 eV, 399.7 eV, and 531.3 eV for both PSPH-Pap and PSPH-Pap-Zn. In the meantime, in the XPS spectra of PSPH-Pap-Zn, there is a strong Zn2p binding energy peak at 1,021.7 eV. This result confirmed the binding between PSPH-Pap and zinc.

### *In vitro* Gastrointestinal Stability of PSPH and PSPH-Zn

The binding between zinc and peptides is reversible. The existence of acidic groups such as -OH, -COOH, -SH, and -NOH may induce the dissociation between zinc and peptides. The human stomach has a low pH around 0.9–1.5. Hence, it is of prime importance to evaluate the gastric stability PSPH-Zn. A desirable zinc chelating peptide should be relatively resistant to the cleavage during gastric digestion, avoiding the release of zinc to form precipitates with phytates or dietary fiber in diets. In the small intestine, minerals such as zinc are transported by transcellular or paracellular pathways. Some non-specific transporters may carry any mineral presented. Hence this transport may be affected by the presence of other metal ions. Minerals supplemented in peptide complexes can be transported differently without potential competition from other metal ions. As shown in Table 2, the gastric digestion stability of PSPH-Pap-Zn was 90.06 ± 1.64%, higher than that of PSPH-Try-Zn 83.23 ± 1.21%. The stabilities of zinc after both gastric and intestinal digestion were 52.99 ± 1.46% for PSPH-Pap-Zn, 33.23 ± 6.71% for PSPH-Try-Zn, both higher than 17.40 ± 5.15% for zinc salts. This may be caused by higher binding capacity of PSPH-Pap than that of PSPH-Try, preventing zinc from dissociating from the complex and dissolving in gastric juice. Overall, our result showed that PSPH derived zinc chelating peptides had better gastrointestinal stability than zinc sulfate and may be used as potential zinc fortifier.

**Table 2:** The *in vitro* gastrointestinal stability of PAPH-Pap, PSPH-Try, and ZnSO_4_·7H_2_O.

	**Gastric digestion stability (%)**	**Gastrointestinal digestion stability (%)**
PSPH-Pap	90.06 ± 1.64^c^	52.99 ± 1.46^c^
PSPH-Try	83.23 ± 1.21^b^	33.23 ± 6.71^b^
ZnSO_4_·7H_2_0	71.09 ± 2.63^a^	17.40 ± 5.15^a^

*Data with different letters represent significantly different mean values with P < 0.05*.

## Conclusions

In this study, five enzymes were used to prepare PSPHs. The PSPHs were characterized for their molecular weight distribution, average particle size, zeta potential, amino acid composition, and zinc binding capacity. Representative samples were analyzed using ATR-FTIR, SEM, and XPS to quantitatively characterize the binding between zinc and PSPH. Our result showed that papain may be used as an enzyme to prepare PSPH. The obtained PSPH-Pap had the average molecular weight around 3,312 Da. It has the highest zinc binding amino acid content and showed the highest zinc binding capacity. PSPH-Pap can retain more than 50% of zinc after *in vitro* gastrointestinal digestion. This study can provide a preliminary knowledge on the application of pumpkin seed in zinc fortification. Future studies should focus on the purification and identification of individual peptide that binds to zinc, while at the same time showing excellent stability in gastrointestinal tract. Efforts should also be made to elucidate the specific binding mode between zinc and this particular peptide, which will enable further developing of zinc supplements with high bioavailability.

## Data Availability Statement

The raw data supporting the conclusions of this article will be made available by the authors, without undue reservation.

## Author Contributions

DL: conceptualization, Investigation, Methodology, Software, Data curation, writing—original draft, writing—review & editing. MP and MY: investigation. BJ: formal analysis, writing—review & editing. HW: writing—review & editing. JC: project administration, funding acquisition, writing—review & editing. All authors contributed to the article and approved the submitted version.

## Conflict of Interest

The authors declare that the research was conducted in the absence of any commercial or financial relationships that could be construed as a potential conflict of interest.
